# Detection of Circulating Tumor Cells in Renal Cell Carcinoma: Disease Stage Correlation and Molecular Characterization

**DOI:** 10.3390/jcm9051372

**Published:** 2020-05-07

**Authors:** Petr Klezl, Eliska Pospisilova, Katarina Kolostova, Jindrich Sonsky, Ondrej Maly, Robert Grill, Ireneusz Pawlak, Vladimir Bobek

**Affiliations:** 1 Department of Urology, University Hospital Kralovske Vinohrady, 3rd Faculty of Medicine Charles University in Prague, Srobarova 50, 10034 Prague, Czech Republic; klezl@volny.cz (P.K.); eliska.pospisilova@outlook.cz (E.P.); jindrich.sonsky@fnkv.cz (J.S.); robert.grill@fnkv.cz (R.G.); 2 Department of Laboratory Genetics, University Hospital Kralovske Vinohrady, Srobarova 50, 10034 Prague, Czech Republic; katarina.kolostova@gmail.com (K.K.); ondmaly@gmail.com (O.M.); 3 Department of Thoracic Surgery, Lower Silesian Cancer Center, Wroclaw, Plac Ludwika Hirszfelda 12, 53-413 Wrocław, Poland; ireneusz.pawlak@dco.com.pl; 4 3rd Department of Surgery University Hospital Motol, 1st Faculty of Medicine Charles University, V Uvalu 84, 150 06 Prague, Czech Republic; 5 Department of Histology and Embryology, Wroclaw Medical University, Wybrzeże Ludwika Pasteura 1, 50-367 Wrocław, Poland; 6 Department of Thoracic Surgery, Masaryk´s Hospital, Krajska zdravotni a.s., Socialni pece 3316/12A, 40113 Usti nad Labem, Czech Republic

**Keywords:** CTCs, renal cancer, culturing, in vitro, MetaCell, gene expression, immunotherapy, PDL-1

## Abstract

The presence of circulating tumor cells (CTCs) in patients with solid tumors is associated with poor prognosis. However, there are limited data concerning the detection of CTCs in renal cell cancer (RCC). The aim of this study is to evaluate the presence of CTCs in peripheral blood of patients with RCC undergoing surgery (n = 186). CTCs were tested before and after surgery as well as during the follow-up period afterwards. In total 495 CTC testing in duplicates were provided. To enrich CTCs, a size-based separation protocol and tube MetaCell® was used. CTCs presence was evaluated by single cell cytomorphology based on vital fluorescence microscopy. Additionally, to standardly applied fluorescence stains, CTCs viability was controlled by mitochondrial activity. CTCs were detected independently on the sampling order in up to 86.7% of the tested blood samples in patients undergoing RCC surgery. There is higher probability of CTC detection with growing tumor size, especially in clear cell renal cell cancer (ccRCC) cases. Similarly, the tumor size corresponds with metastasis presence and lymph node positivity and CTC detection. This paper describes for the first-time successful analysis of viable CTCs and their mitochondria as a part of the functional characterization of CTCs in RCC.

## 1. Introduction

Radical or partial nephrectomy remains the only curative treatment for RCC. More than 30% of localized renal cell carcinoma (RCC) recur or metastasize after surgery [1]. RCC is a paradigm of chemoresistant cancer. Even in combination with immunotherapy, chemotherapy is associated with low response rates [2,3]. RCC is a highly invasive cancer which could benefit from non-invasive biomarkers for early diagnosis and disease monitoring. Liquid biopsy, in particular the circulating tumor cells (CTCs), offer a promising tool for assessment of the tumor burden and tumor invasion capability.

CTCs are an undeniable part of a connection between primary tumors and secondary metastatic sites. The CTC examination belongs to the group of liquid biopsy tests, which provide more detailed information on heterogeneity of tumor disease in patients, under minimally invasive procedures (e.g., blood withdrawal). 

The CTCs represent a possible tool in monitoring this kind of tumor evolution in time. As defined in liquid dynamic medicine [4], where a patient is a control subject to himself during the treatment, we may evaluate the cancer cell changes enabling personalization of oncological therapy.

The main target of the presented study is to monitor CTC presence in patients with renal cell carcinoma undergoing surgery. Next, cytomorphology of CTCs was evaluated by means of vital fluorescence microscopy with a special focus on mitochondrial network evaluation.

Since complete removal of metastatic lesions has a significant potential to improve prognosis of RCC, we believe CTCs could be a helpful tool in reassessing metastasis infiltration in patients with advanced form of the disease.

**Figure 1:**
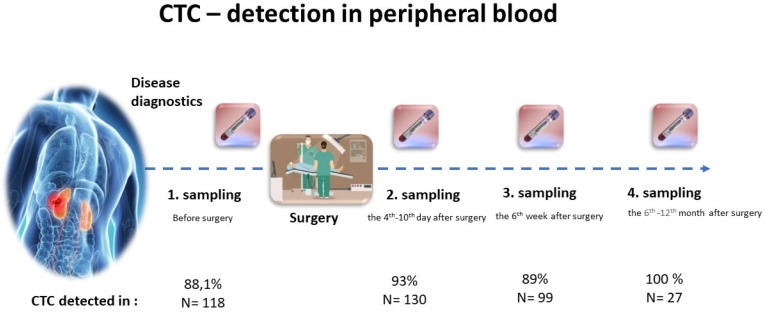
Schedule of blood collection.

We believe that future molecular analysis of CTCs may support progress in RCC treatment, covering multiple aspects of RCC biology as well in blood as in tissue. CTCs may become a part of clinical studies testing efficiency of new drug treatment (Figure 1).

## 2. Materials and Methods

Total number of 186 patients (*n* = 186) who underwent surgical renal resection because of tumor mass presence were included into the study in 2016–2018. Peripheral blood (2 × 8 mL, EDTA) was taken in several time points: 1. before surgery, 2. within 24 hours after surgery, 3. at 2-week follow-up, 4. at follow-up every 6 months; between 2 and 4 blood withdrawals were done per patient. In total 495 CTCs tests were performed. The CTCs test is based on duplicates evaluation. 

To enrich CTCs a size-based separation protocol and tube MetaCell® was used [5,6,7,8]. CTC presence is evaluated by single cell cytomorphology, which could be followed by molecular testing (e.g., qPCR analysis, sequencing) or standard immunohistochemistry. Total of 186 patients diagnosed with RCC have been enrolled in the study in accordance with the Declaration of Helsinki and ethical committee approval was granted. All patients were candidates for surgical treatment. Informed consent was obtained from each patient before any clinical data were collected. The patient characteristics are shown in Table 1. For each patient, approximately 2 × 8 mL of venous blood was drawn from the cubital veins and placed into S-Monovette tubes (Sarstedt AG & Co., Numbrecht, Germany) containing 1.6 mg EDTA/mL blood as an anticoagulant. The samples were processed at room temperature using an isolation procedure completed within 24 hours after the blood draw.

### 2.1. CTCs Enrichment and Culture

A size-based separation method for viable CTC-enrichment from peripheral blood was used (MetaCell®, MetaCell s.r.o., Ostrava, Czech Republic) [3,4,5]. 

The complex of separation membrane filter, which is kept in a plastic ring, was transferred directly with enriched cells into a 6-well culture plate. Total of 4 mL RPMI media is added to the filter top and enriched cells are cultured on the membrane in vitro under standard cell culture conditions (37 °C, 5% CO2 atmosphere) and observed using an inverted microscope. CTCs are grown in FBS-enriched RPMI medium (10%) for a period of minimum 3–5 days. A microscopic slide was placed under the separation membrane and CTCs may naturally grow invasively and set up new cell colonies on the microscopic slide. A microscopic slide culture is preferred if immunohistochemistry/immunofluorescence analysis is planned. 

### 2.2. Cytomorphological Analysis

The viable cells stained by vital fluorescence stains (Celltracker, NucBleu, Mitotracker, Thermofisher Scientific, Waltham, MA, USA) were examined using fluorescence microscopy in three steps: (i) Screening at x20 magnification to locate the cells, (ii) observation at x40 magnification for detailed cytomorphological analysis. Isolated cells and/or clusters of cells of interest (immunostained or not) were selected, digitized, and examined by an experienced researcher and/or pathologist. (iii) Evaluate the CTCs—number CTCs were defined as cells presenting the following characteristics: (a) Nuclear size equal to or larger than 15 μm; (b) irregularity of the nuclear contour; (c) presence of a visible cytoplasm and the size of the cell; (d) presence of prominent nucleoli and their number; (e) high nuclear-cytoplasmatic ratio. The nuclear-cytoplasmatic (N/C) ratio is evaluated with respect to the different morphology of growing cells in comparison to the N/C ratio in tissue samples. 

### 2.3. Immunohistochemistry Analysis

Based on the standard immunohistochemistry protocols, cytokeratin and vimentin presence was tested in cells captured on the membranes. The membranes were fixed by air first at least for 24 hours. As next step, the immuno-histochemistry protocol was used as follows. Two antibody clones were used, pancytokeratin (SigmaAldrich, St. Louis, MO, USA) and vimentin (Sigma Aldrich, St. Louis, MO, USA) 

## 3. Results

### 3.1. CTCs Prevalence 

**Figure 2:**
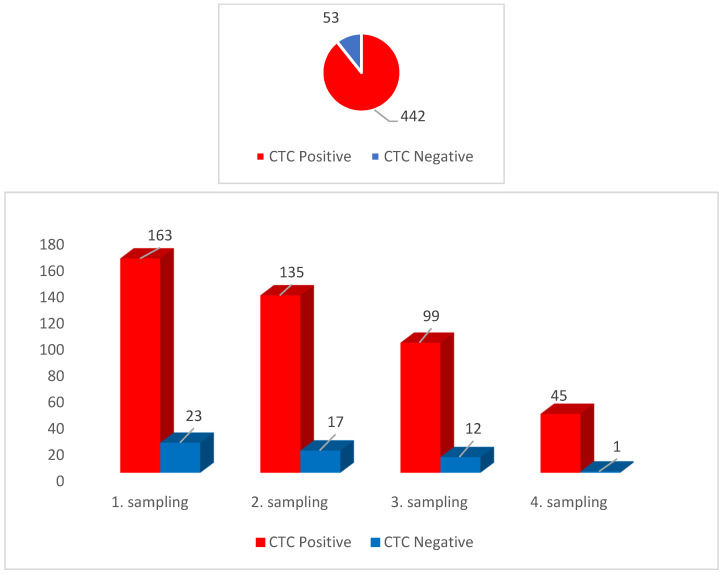
Circulating tumor cells (CTCs) detected in renal cell cancer (RCC) patients for one-year follow-up in four blood samplings. All together 495 samples (in duplicates) were evaluated, with an average 86.6% CTC positivity rate.

**Figure 3:**
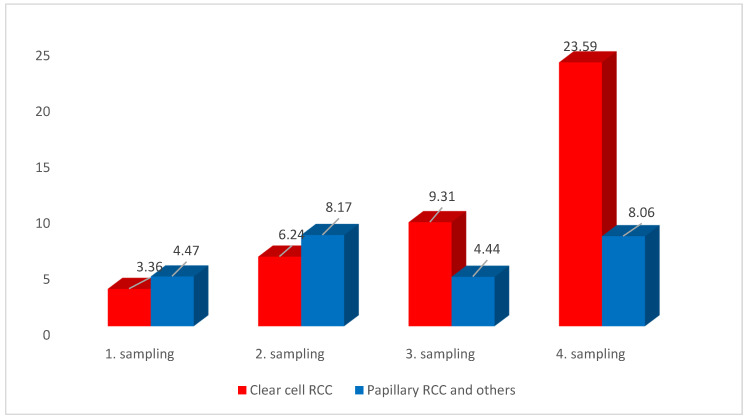
CTC—number (average per 1 mL of blood) detected in RCC patients for one-year follow-up in four blood samplings showing differences in CTC load between ccRCC and other RC types.

CTCs were detected independently on the sampling order in up to 86.7% of the tested blood samples (495) in patients undergoing surgery (Figure 2). CTCs were detected in patients harboring tumors with different histology origins, showing that the average numbers of CTCs are being slightly higher in clear cell RCC (ccRCC) several months after surgery, if compared with papillary RCC (Figure 3). 

Altogether 270 samples were tested as positive in ccRCC, 36 samples were tested as positive in papillary RCC and 30 samples in other RCC types. 

### 3.2. CTCs Cytomorphological Parameters

Cytomorphological analysis of CTCs consisted of two microscopy evaluations, in two different time points (the day 3 and 5 after enrichment and in vitro culture), provided by two independent evaluators. 

The average CTCs size for the groups of ccRCC is 24 ± 6.3, the average CTCs size for the group of papillary renal cell cancer (pRCC) is 22 ± 3.4, and for the other types is 27 ± 3.7 µm. 

**Figure 4:**
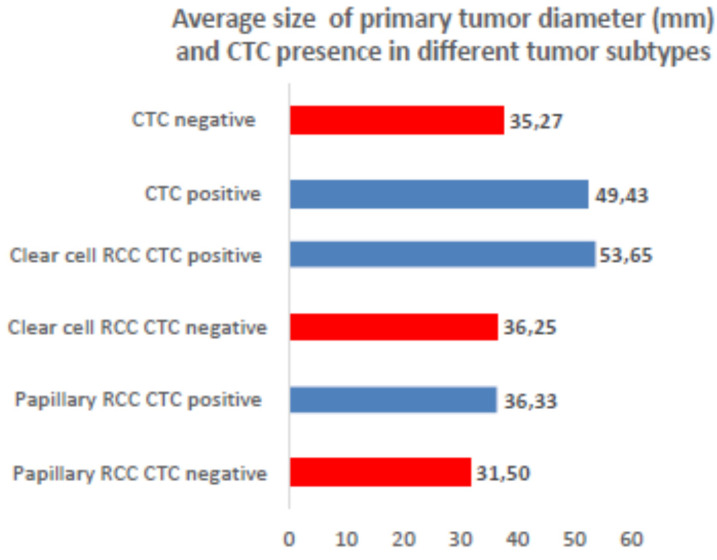
Relationships between tumor size and CTC positivity. The largest tumor diameter and higher CTC number were detected in RCC.

The average CTC number doubled between the day 3 and 5, which corresponds to the expectations of the cell growth speed (1st microscopy evaluation = 4.18 cells, 2nd microscopy evaluation = 7.93 cells). There is higher probability of CTC detection with growing tumor size (Figure 4.), especially in ccRCC cases. The tumor size corresponds with metastasis presence and lymph node positivity and CTC activity. 

The metabolism of CTCs has been evaluated by mitochondria staining, which discovered an interesting mitochondrial network. The network was significantly different from the mitochondrial network observed in cells with a blood origin (e.g., basophils, monocytes, neutrophils).

The blood cells and cancer cells may be easily distinguished by the very sophisticated mitochondrial network. To evaluate the mitochondrial network an automatized Image J evaluation procedure was used. 

Parallel expression of cytokeratin and vimentin was revealed by immuno-histochemistry suggesting an ongoing epithelial mesenchymal transition. 

### 3.3. Gene Epression

**Figure 5:**
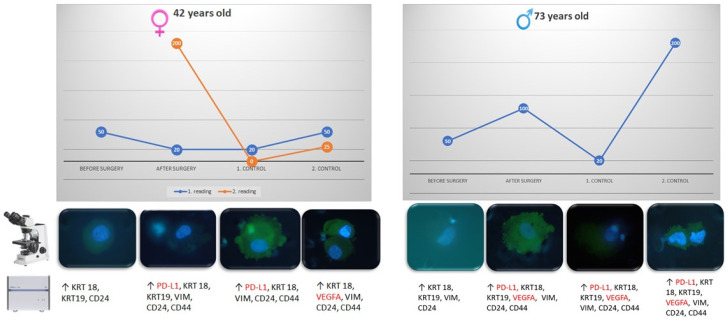
CTC follow-up during one-year period. We demonstrate changes during the four samplings not only in the CTC number (graph lines) and cytomorphology, but also in the gene expression (see elevated genes in CTCs below). Some of the genes with elevated expression could be used as markers to indicate specific therapy targets (e.g., VEGF, PD-L1). PD-L1 and VEGF expressions are shown in red.

**Figure 6:**
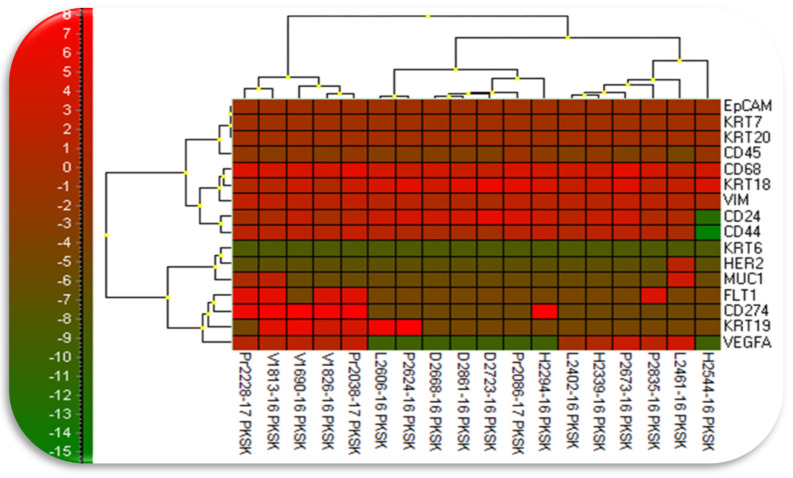
Relative RNA amount displayed in clusters after gene expression analysis using GenEx vs.6–software. (MultiD Analyses AB, Goteborg, Sweden). Clear distinction between CTCs expressing PD-L1 is shown (see arrow-cluster on the left).

**Figure 7:**
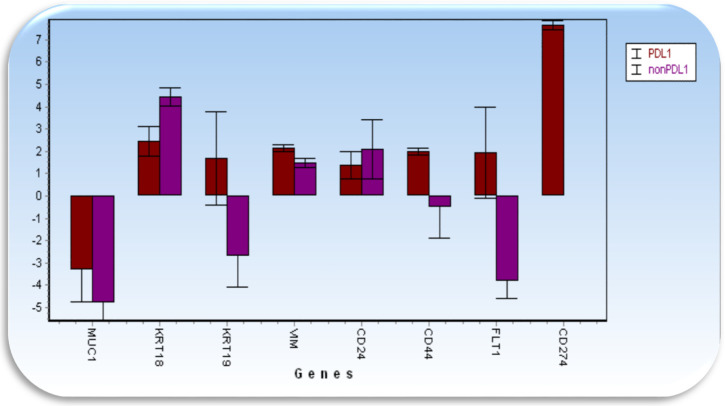
Gene expression analysis of CTC showing differences between the CTC PDL1+ (brown) and CTC PDL1- (violet). The arrows indicate genes expressed differentially between tested groups.

Gene expression analysis revealed elevated gene expression in enriched CTCs for genes KRT18 (keratin 18), VIM (vimentin) when compared with the white blood cell fraction (Figure 5). Further, it showed that the character of CTCs undergoes changes during follow-up period (Figure 6). From the therapeutic point of view the most interesting changes were seen in patients expressing elevated VEGF (vascular endothelial growth factor) and/or PD-L1 (programmed death ligand 1, CD274) on CTCs. VEGF and PD-L1 are shown on follow-up figures (Figure 5 below the graphs and Figure 7).

## 4. Discussion

Only few studies have reported on CTC analysis in patients with RCC [9]. In general, detection of CTCs requires specific techniques able to overcome problems related to identification and isolation of tumors cells from blood. Indeed, there is not any specific marker that allows to uniquely distinguish a CTC from other blood cells, since tumors with different histological and molecular features express diverse patterns of markers, and even a single histological tumor type can present heterogeneous markers. Moreover, given the considerably small number of CTCs possibly present in peripheral blood when compared with the other blood circulating cells, enrichment techniques are necessary to increase the sensitivity to an acceptable level. We believe the observation of size-based enriched cells in a viable stage may help to distinguish the cancerous character of captured cells comparing the standard cytomorphological parameters and additionally mitochondrial network of tumorigenic vs. benign cells. 

Intra-tumoral heterogeneity is a prominent feature of RCC, as evidenced by several groups using multiregional sequencing of RCC tumorous and metastatic tissues compared to sequencing of adjacent normal kidney tissue [10,11,12]. Cancer cells from RCC patients are prone to mesenchymal–epithelial transition (EMT) [13,14,15] and often lack epithelial antigens, which may impair their capture from blood and analysis when epithelial marker-dependent collection/detection methods are used.

For example, CellSearch system, platform only approved by FDA showed a very low detection rate in patients with localized and metastatic RCC [13]. The expression of EMT by RCC cancer cells resulting in a lack of epithelial adhesion molecules could partially explain the failure of this method. 

Significant discrepancies between the number of CTC enumerated by the CellSearch and other filtration method (the ISET systems), were found in patients with lung cancer in a study by Farace et al. [16]. There were 30% of patients who were negative according to CellSearch while only 5% were negative using ISET. Concordant results only concern 47% patients. Similar results were described in study patients with RCC: CTCs were in 13 from 36 patients (36.1%) using ISET and only in 7 from 36 patients (19.4%) using the CellSearch platform [17]. In general, better detection of CTC in patients via filtration methods than CellSearch was documented in a number of studies [18]. The filtration systems may be much more efficient in identifying the circulating cells because CellSearch cannot detect the loss of epithelial markers which is associated with tumor cells undergoing the EMT process.

Our immune-histochemistry data have shown that CTCs from patients with RCC may express cytokeratines as well vimentin. The duality of the filaments confirms the dynamics of the changes found in the tumor masses. 

A successful application of CTCs capture based on EpCAM positive has been very limited so far in metastatic RCC. High plasticity and heterogeneity of CTC morphology challenges currently available enrichment and detection techniques with EpCAM as the usual surface marker being underrepresented in metastatic RCC. The size-based separation method we presented in combination with a vital mitochondria stain enables to characterize non-hematopoietic cells in the peripheral blood stream with varying characteristics and define CTC subgroups that distinctly could be possibly associated with metastatic potential.

In general, early stages of RCC are not associated with clinical manifestation and so the early detection of the disease remains a significant challenge. 

As the RCC is then later seen as a chemo-resistant disease it has to be considered that in the last few years several innovative treatment regimens have been developed for the management of metastatic RCC. In RCC increased understanding of genetics and molecular biology led to successful employment of agents targeting the VEGF and mTOR pathways [19]. But even if different treatment strategies are available, no response predictive markers have been included in clinical practice and so the choice between these different drugs is generally made by considering the clinical outcome, patient’s preference, and toxicity profile of each agent. The resulting plurality of available treatment options is significantly limited by available parameters for a personalized implementation of these agents. We therefore believe testing CTCs profiles by gene expression analysis of the targetable genes, may change and improve RCC therapy outcomes. 

Checkpoint inhibition targeting the programmed cell death 1receptor (PD-1) pathway has become a common treatment across various tumor types. Nivolumab was the first-in-class PD-1 therapy and was approved for the treatment of RCC based on the results of a phase III trial comparing nivolumab with everolimus [20].

Over-expression of PD-L1 has been identified as a pathway that metastatic tumor cells use to evade immune detection. PD-L1 binds to PD-1 on T-cells and suppresses their activity. Immunotherapy based on inhibition of PD-1 or PD-L1 represents a breakthrough in the treatment of advanced cancers. Today, solid tumor patients are screened for PD-L1 expression on their tumor cells following a tissue biopsy. PD-L1 expression levels have been demonstrated to be a reasonable biomarker for stratifying patients that will respond better to immunotherapy.

Utilizing biopsies for this patient stratification does have its limitations. Specifically, tumor biopsies are risky, expensive, and cannot be performed serially to understand cancer status throughout the course of a treatment. Liquid biopsy may possibly illustrate disease control because it is minimally invasive and can easily be repeated. Unfortunately, low specificity is the biggest disadvantage of the presented size-based separation method. Second advantage is high sensitivity and isolation of viable cells with subsequent culturing. The isolation of whole cells provides further possibility of molecular characterization by specification of cells.

Measuring PD-L1 expression on CTCs through a simple blood draw represents a low risk approach to initial patient stratification with minimal cost and gives us an opportunity to monitor PD-L1 expression of patient tumor cells over the course of the disease.

## Figures and Tables

**Table 1:** Patient characteristics.

All			Primary Tumor Diameter (mm)							Total			
**All**			49.3							168			
**CTC positive**			50.08							137			
**CTC negative**			34.9							20			
**Not evaluable**			65.81							11			
**CTC**		**CTC Positive**				**CTC Negative**			**Not evaluable**			**Total Samples**	**Patients**
		**Patients**		**In %**		**Patients**		**In %**					
**Clear cell RCC**		270		85.99		33		10.51	11			314	119
**Papillary RCC**		56		84.85		7		10.61	3			66	22
**Clear cell RCC and Papillary RCC**		11		84.62		2		15.38				13	4
**Chromophobe RCC**		19		76.00		4		16.00	2			25	8
**CTC**	**Type**				**CTC Positive**			**CTC Negative**			**Not Evaluable**		**Total**
					**Patients**		**In %**	**Patients**	**In %**				
1. sampling	**Clear cell RCC**				21		75.00	5	17.86		2		28
1. sampling	**Papillary RCC and others**				13		68.42	4	21.05		2		19
2. sampling	**Clear cell RCC**				27		96.43	1	3.57				28
2. sampling	**Papillary RCC and others**				18		94.74	1	5.26				19
3. sampling	**Clear cell RCC**				24		85.71	4	14.29				28
3. sampling	**Papillary RCC and others**				18		94.74				1		19
4. sampling	**Clear cell RCC**				28		100.00						28
4. sampling	**Papillary RCC and others**				18		94.74	1	5.26				19
